# Actin Polymerization Controls the Organization of WASH Domains at the Surface of Endosomes

**DOI:** 10.1371/journal.pone.0039774

**Published:** 2012-06-21

**Authors:** Emmanuel Derivery, Emmanuèle Helfer, Véronique Henriot, Alexis Gautreau

**Affiliations:** Laboratoire d'Enzymologie et Biochimie Structurales, CNRS UPR3082, Gif-sur-Yvette, France; Thomas Jefferson University, United States of America

## Abstract

Sorting of cargoes in endosomes occurs through their selective enrichment into sorting platforms, where transport intermediates are generated. The WASH complex, which directly binds to lipids, activates the Arp2/3 complex and hence actin polymerization onto such sorting platforms. Here, we analyzed the role of actin polymerization in the physiology of endosomal domains containing WASH using quantitative image analysis. Actin depolymerization is known to enlarge endosomes. Using a novel colocalization method that is insensitive to the heterogeneity of size and shape of endosomes, we further show that preventing the generation of branched actin networks induces endosomal accumulation of the WASH complex. Moreover, we found that actin depolymerization induces a dramatic decrease in the recovery of endosomal WASH after photobleaching. This result suggests a built-in turnover, where the actin network, i.e. the product of the WASH complex, contributes to the dynamic exchange of the WASH complex by promoting its detachment from endosomes. Our experiments also provide evidence for a role of actin polymerization in the lateral compartmentalization of endosomes: several WASH domains exist at the surface of enlarged endosomes, however, the WASH domains coalesce upon actin depolymerization or Arp2/3 depletion. Branched actin networks are thus involved in the regulation of the size of WASH domains. The potential role of this regulation in membrane scission are discussed.

## Introduction

The Arp2/3 complex is a major actin nucleator that generates branched actin networks at different cellular locations [1]. Recent evidence suggest a division of labor between distinct Arp2/3 activators [2], [3], [4], [5]. WAVE proteins activate the Arp2/3 complex at the plasma membrane and generate lamellipodia. N-WASP generates a burst of actin polymerization at coated pits during clathrin mediated endocytosis. WHAMM regulates Golgi morphology and ER to Golgi transport [6]. We and others recently reported that WASH is found at the surface of vesicles of the endosomal/lysosomal system, yet with a marked enrichment in sorting endosomes [7], [8], [9], [10], [11], [12]. Consistently, WASH regulates endosome morphology and WASH depletion affects the major endosomal routes, including recycling, degradation and retrograde pathways [7], [8], [9], [10]. WASH has also been implicated in removal of v-ATPases from lysosomes [11]. The division of labor between Arp2/3 activators is, however, not absolute. For example, N-WASP was also detected at the rear of endosomes that are propelled in the cytoplasm through actin comet tails in special cases, when cells were stimulated by phorbol esters or when the lipid phosphatidylinositol(4,5)bis-phosphate was overproduced [13], [14]. However, in normal circumstances, endosomes move along microtubules and WASH appears as the major Arp2/3 activator at their surface [7]. Regulation of actin polymerization at the surface of endosomes also implicates Annexin A2 [15] and diaphanous related formins [16], [17].

Actin polymerization at the surface of endosomes was previously involved in the regulation of vesicle fusion [18], [19], [20], [21] and in the biogenesis of multivesicular bodies [15]. WASH dependent branched actin networks, specifically labeled with the branch marker, cortactin [22], were shown to define specific domains of the endosomal membrane [7], [8], [9], [23]. These domains might be due in part to the lipid binding ability of the multiprotein complex that WASH forms [7], [24]. These actin rich endosomal domains were found to be important for cargo sorting [25]. Branched actin networks at the surface of endosomes are important in at least two steps. First, they stabilize a membrane tubule, while it clusters cargoes [23]. Then, they promote the scission of the transport intermediate through an incompletely understood mechanism that involves dynamin [7]. The latter step appears analogous to the collaboration between dynamin and the N-WASP dependent branched actin network during scission of the clathrin coated pit [26], [27].

The identity of the endosomal transport intermediate, containing specific cargoes, is conferred by its content in Rab GTPases that defines its target destination [28], [29]. Lateral compartmentalization of cargoes and Rab proteins relies on a dynamic network of lipid-protein interactions [30], [31]. For example, Rab5 domains are generated at the surface of early endosomes through the recruitment of several Rab5 effectors, which oligomerize, bind to lipids and further activate Rab5 [32], [33], [34], [35]. These dynamic proteo-lipidic domains contrast with membrane rafts that depend on strong hydrophobic lipid-lipid interactions [36]. Such dynamic proteo-lipidic domains were proposed to depend on cytoskeletal elements, among other factors, for their cohesion [28].

**Figure 1:**
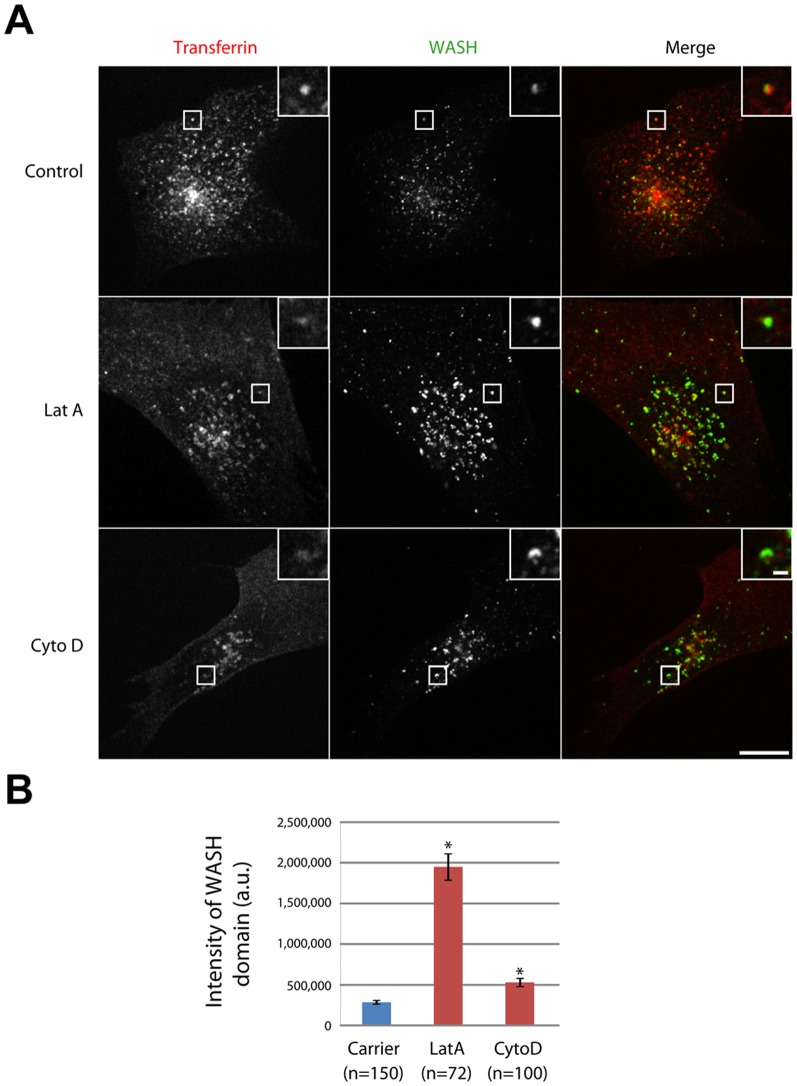
Actin depolymerization increases the amount of endosomal WASH. (A) 3T3 cells were loaded with fluorescent Transferrin (Tf) until equilibrium, then treated with 0.2 µM Latrunculin A (LatA) for 10 min, 1 µM CytochalasinD (CytoD) for 30 min, or carrier in the continuous presence of Tf. Cells were processed for immunofluorescence using WASH antibody and observed by spinning disk confocal microscopy. A single plane is displayed. Scale bar: 10 µm (1 µm in inserts). LatA and CytoD treatments increase the fluorescence signal of WASH. (B) Since endosomes are clustered in the perinuclear region, the increase of WASH intensity was quantified on isolated endosomes from the cell periphery (see Methods). Both LatA and CytoD treatments induce a statistically significant increase of the WASH fluorescence signal (n refers to the number of endosomes, *: p<0.001, one way ANOVA followed by a Tukey pairwise comparison).

**Figure 2:**
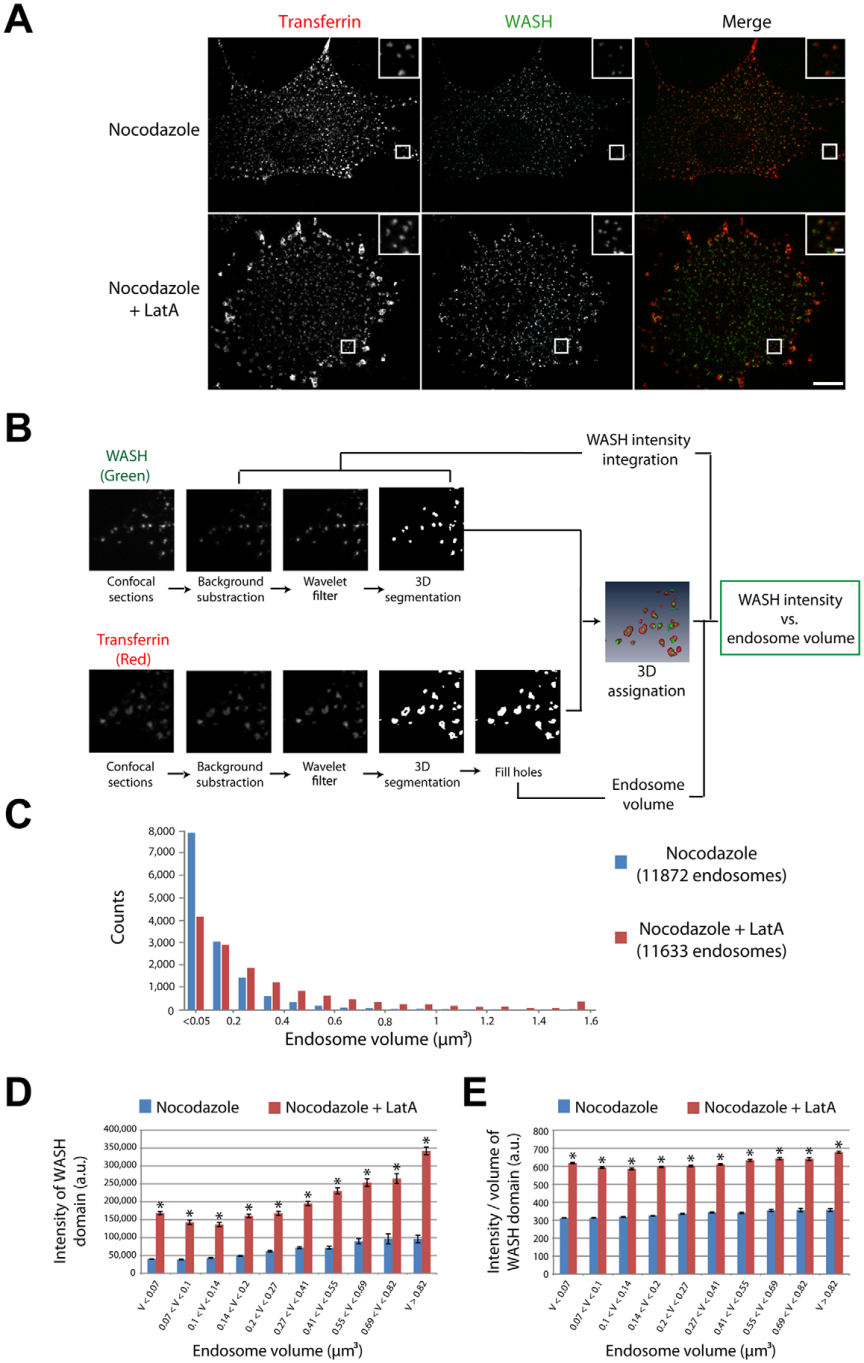
Automated quantification of the increase of endosomal WASH upon actin depolymerization. (A) 3T3 cells were loaded with fluorescent Tf until equilibrium, then treated with 10 µM nocodazole in the continuous presence of Tf for 1 h, then treated with 0.2 µM LatA or carrier in the presence of nocodazole and Tf for 10 min. Cells were then processed for immunofluorescence and observed as in Fig. 1. Scale bar: 10 µm (1 µm in inserts). The nocodazole treatment induces scattering of the endosomes thus allowing detection of the whole population of endosomes. The LatA treatment enlarges endosomes and increases the intensity of WASH on endosomes. (B) Image processing workflow (see also Methods). WASH and Tf images were processed as in Fig. 1. As the endosomes were scattered due to nocodazole treatment, both Tf-positive endosomes and WASH domains could be detected automatically after 3D segmentation, then each WASH domain was assigned to its proper endosome using a custom-made program (see Methods) and the WASH intensity was measured on each endosome. (C) Image stacks of cells treated as in Fig. 2A were segmented in 3D for both channels and analyzed (24 cells, 11872 endosomes for control; 44 cells, 11633 endosomes for LatA). The LatA treatment induces a shift of endosome sizes towards larger volumes (p<0.001, χ^2^ test). (D) The average intensity (± s.e.m.) of WASH domains increases with endosome size in both control and LatA-treated cells. Moreover, within a given endosome volume range, actin depolymerization induces an increase of WASH intensity as compared to non-treated cells. (E) The average ‘apparent concentration’ of WASH (± s.e.m.) in domains increases when actin is depolymerized and is independent of endosome volume. All data were analyzed by a two way ANOVA using treatments and volume bins as parameters, and pairewise comparisons were performed using a Tukey test. *: p<0.001 compared with control within the same volume bin.

Here we investigated the role of actin polymerization at the surface of endosomes by monitoring the WASH marker, which defines an endosomal domain. Using quantitative image analysis, we identified two specific feedback regulations of the WASH domain by the branched actin network it nucleates: actin filaments control the lateral organization of WASH domains and the dynamic exchange of WASH molecules between the endosomal domain and the cytosolic pool. The potential role of these feedback regulations in the mechanism of endosomal scission is discussed.

## Results

### Actin dynamics promote dynamic exchange of the WASH complex between endosomal and cytosolic pools

To analyze the role of actin dynamics in the physiology of endosomal WASH domains, 3T3 cells were loaded with fluorescent transferrin (Tf) to label sorting and recycling endosomes, then treated with actin depolymerizing drugs in the presence of Tf. We used Latrunculin A (LatA), which sequesters monomeric actin and prevents it from polymerizing [37], and Cytochalasin D (CytoD), which caps filament barbed ends [38]. Both treatments seemed to induce an increase of the WASH fluorescence signal on endosomes as compared to the control (Fig. 1A). We thus decided to quantify the WASH signal on endosomes in presence or absence of actin. Endosomes were detected using the Tf channel and the fluorescence intensity of the corresponding WASH domains was measured (see Methods). The high degree of endosome clustering in the perinuclear region restricted this observation to the few endosomes that are scattered in the cytoplasm. This preliminary analysis showed that both treatments induce a statistically significant increase of the WASH signal per endosome (Fig. 1B). However the effect of CytoD is less pronounced than that of LatA, consistent with the fact that actin disassembly by CytoD is less complete than the one induced by LatA. These results suggest that actin dynamics play a role in the recruitment of WASH onto endosomes.

**Figure 3:**
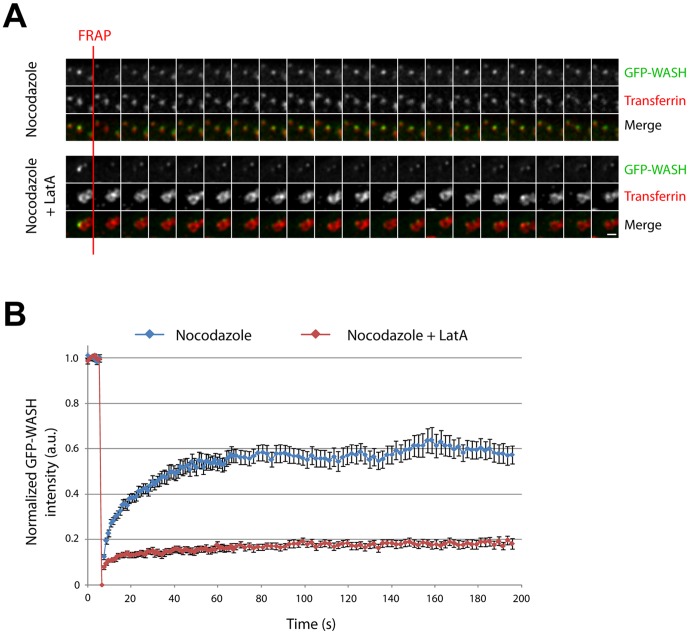
Actin depolymerization inhibits WASH recovery on endosomes after photobleaching. (A) Stable 3T3 cells expressing GFP-WASH were treated as in Fig. 2A. WASH domains were photobleached and signal recovery was monitored for 3 min. A representative sequence of recovery is displayed (8 sec between frames). Scale bar: 1 µm. (B) Recovery of 72 WASH domains for 16 control cells and 98 WASH domains for 13 LatA-treated cells were pooled from 2 independent experiments to calculate the average (± s.e.m.) of WASH intensity at each time point.

**Figure 4:**
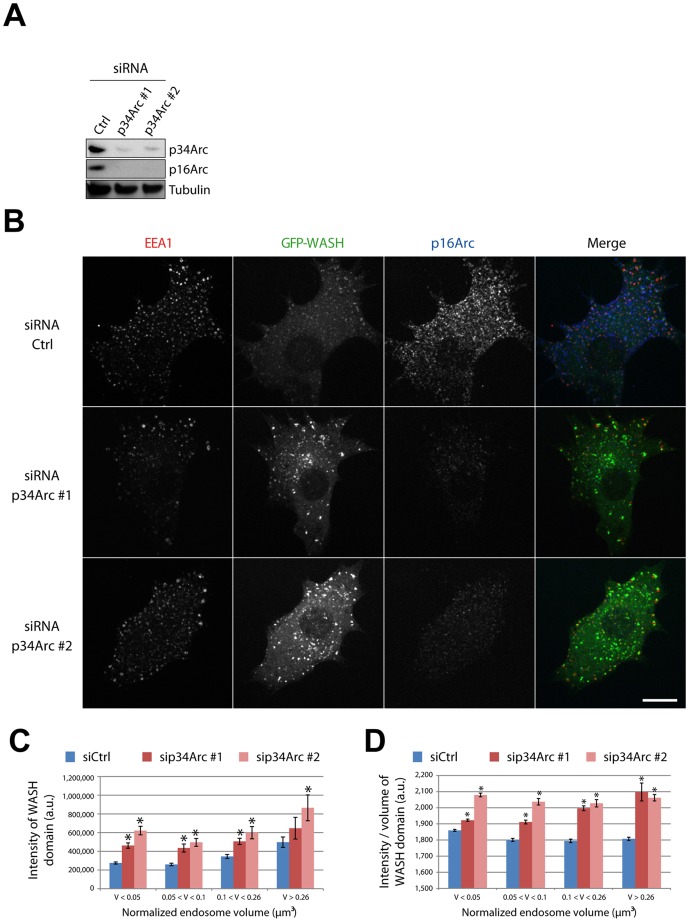
Depletion of the Arp2/3 complex increases the amount of endosomal WASH. (A) Stable 3T3 cells expressing GFP-WASH were depleted from the Arp2/3 complex using siRNAs and analyzed by Western Blot. (B) siRNA-transfected cells were treated with 10 µM nocodazole for 1 h, then processed for immunofluorescence using antibodies recognizing EEA1 and the p16Arc subunit of the Arp2/3 complex, and observed by spinning disk confocal microscopy (single planes). Scale bar: 10 µm. Arp2/3 depletion increases endosomal WASH staining. (C–D) Image stacks (36 cells, 6046 endosomes for Ctrl siRNA; 31 cells, 3889 endosomes for p34Arc #1 siRNA; 26 cells, 3470 endosomes for p34Arc #2 siRNA) were processed and presented as in Fig. 2D–E after normalization of endosomes volumes. Upon Arp2/3 complex depletion, the intensity and ‘apparent concentration’ of GFP-WASH domains increases but does not depend on endosome volume. *: p<0.001 compared with control within the same volume bin, two way ANOVA using treatments and volume bins as parameters, followed by pairwise comparisons using Tukey test.

To confirm this result more quantitatively, we needed an accurate statistical analysis to measure precisely the WASH signal on each endosome. This obviously required the accumulation of data over thousands of endosomes, due to their high heterogeneity in size and shape. We thus additionally treated the cells with nocodazole, which depolymerizes microtubules and therefore prevents endosome clustering around the nucleus. In these conditions, we were able to resolve almost all endosomes. In these conditions as well, the WASH signal appeared brighter in LatA-treated cells than in control cells (Fig. 2A). We verified that branched actin networks were depolymerized upon LatA treatment (Fig. S1).

**Figure 5:**
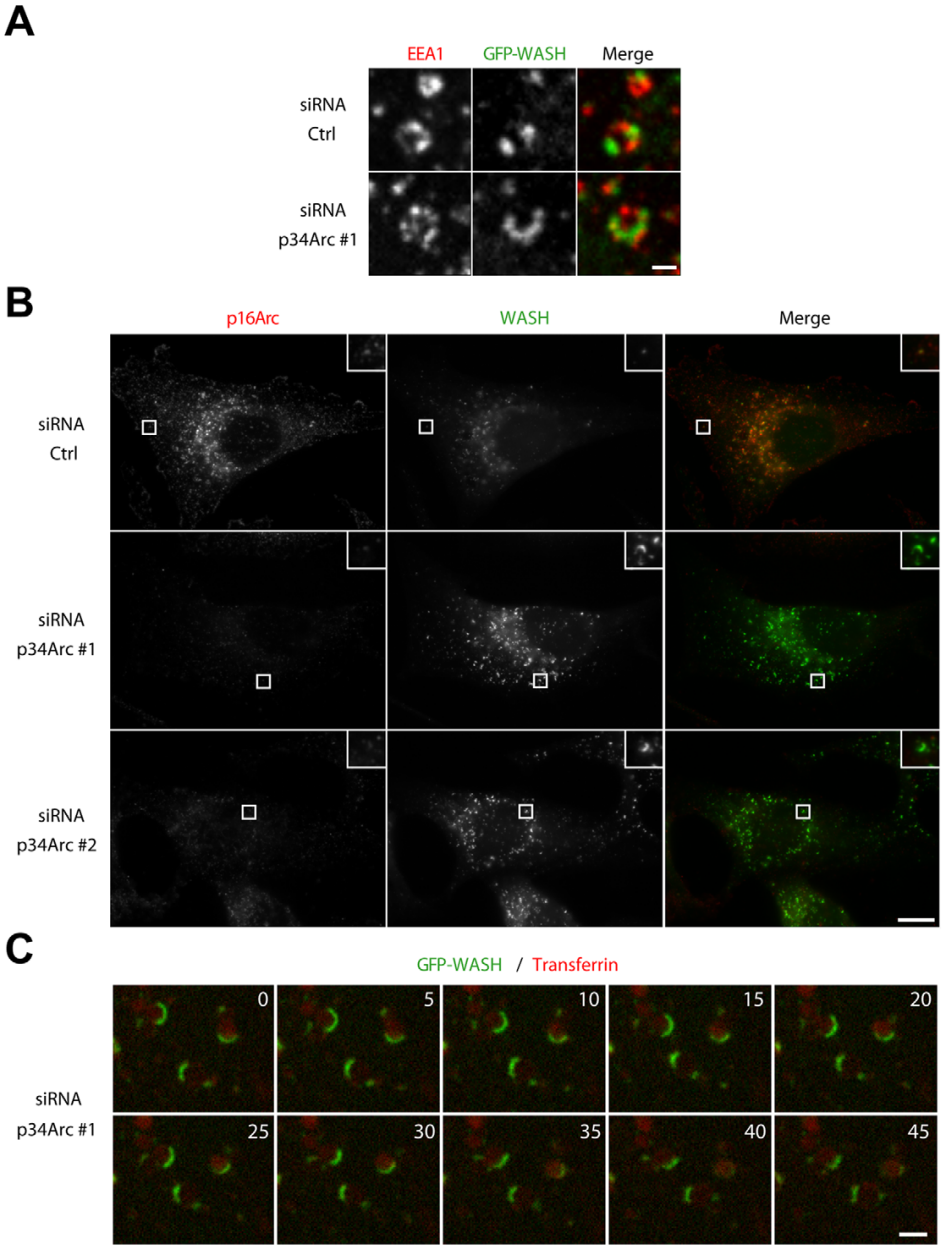
Arp2/3 depletion induces the appearance of elongated WASH domains. (A) Stable 3T3 cells expressing GFP-WASH were depleted of the Arp2/3 complex and treated with nocodazole as in Fig. 4B. Scale bar: 1 µm. GFP-WASH forms a crescent at the surface of large EEA1 positive endosomes upon Arp2/3 depletion. (B) 3T3 cells were depleted of the Arp2/3 complex and processed for immunofluorescence using WASH and p16Arc antibodies. Scale bar: 10 µm. WASH crescents are also observed with endogenous WASH in the absence of nocodazole. (C) Stable 3T3 cells expressing GFP-WASH depleted for the Arp2/3 complex and imaged by live spinning disk confocal microscopy after Tf loading. Elapsed time is in seconds. Scale bar: 2 µm. Arp2/3 depletion inhibits Tf uptake and WASH forms crescents at the surface of large endosomes that are dimly stained by Tf.

**Figure 6:**
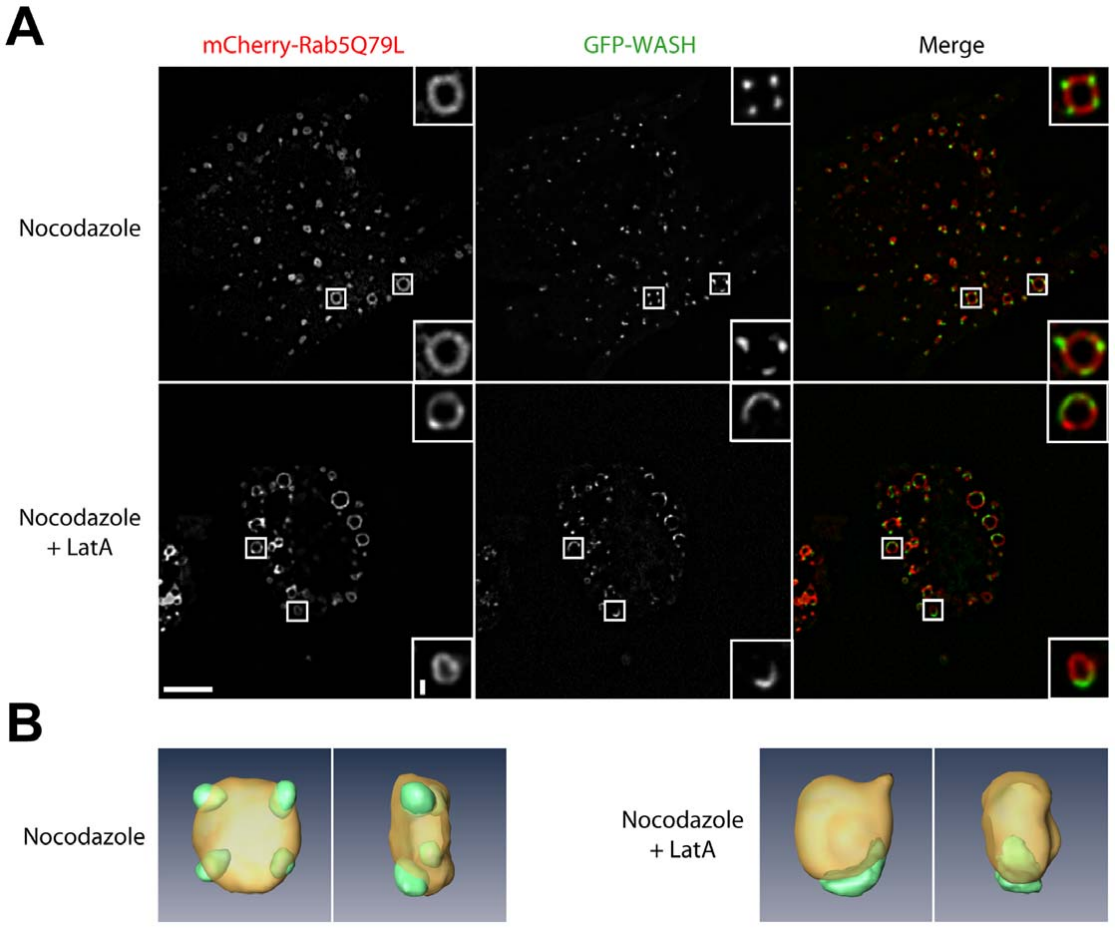
Actin depolymerization induces coalescence of WASH domains. (A) Stable 3T3 cells expressing GFP-WASH were transfected with mCherry-Rab5Q79L to enlarge endosomes and treated with nocodozale and LatA as in Fig. 2A. Cells were imaged by spinning disk confocal microscopy (single planes). Scale bar: 10 µm (1 µm in inserts). WASH localizes to discrete domains at the surface of enlarged endosomes in control cells, but to a unique, crescent shaped structure upon actin depolymerization. (B) Confocal z-sections of the endosomes shown in the top insets in (A) were processed for 3D reconstruction (front and side views).

Upon LatA treatment, endosomes appeared larger, consistently with previous observations [9], [15]. We thus wondered if the increase of WASH intensity was a simple consequence of endosome enlargement. We developed an automated procedure to detect Tf-positive endosomes and WASH domains from confocal z-stacks of cells, which allowed us to measure the intensity of each WASH domain as well as the volume of each endosome (Fig. 2B). We then faced the problem of assigning automatically each WASH domain to its respective endosome. Classical object-based colocalization methods associate objects segmented in two different channels when the distance between their fluorescence centroids is below a threshold limit, usually fixed to the resolution of the microscope [39]. However, classical object-based methods perform poorly in our case, especially on LatA enlarged endosomes, because the cytoplasm is crowded with endosomes and because WASH localizes to domains, which may be far from the centroid of large endosomes. Increasing the threshold did not alleviate the problem and still yielded false assignations. We thus developed a novel object-based colocalization method based on a local, rather than global, threshold to account for the large heterogeneity of endosome size and shape. The principle of this method is the following (see Methods for further details): 1) a yellow stack composed of common pixels is generated from the segmented images of both green and red channels; 2) this stack is then segmented and each yellow region is assigned to its correct red and green regions, from which the association between the red and the green regions is deduced. Although a common pixel criteria is not usually a good marker of colocalization because of the limited resolution of the microscope, the probability to have more than one endosome per diffraction limited volume is strongly diminished in our case due to the use of nocodazole to scatter compartments. This method resulted in reliable 3D-assignations between endosomes and WASH domains, independently on their size and shape, and thus allowed us to quantitatively address the relationship between WASH intensity and endosome volume in the presence or not of LatA.

**Figure 7:**
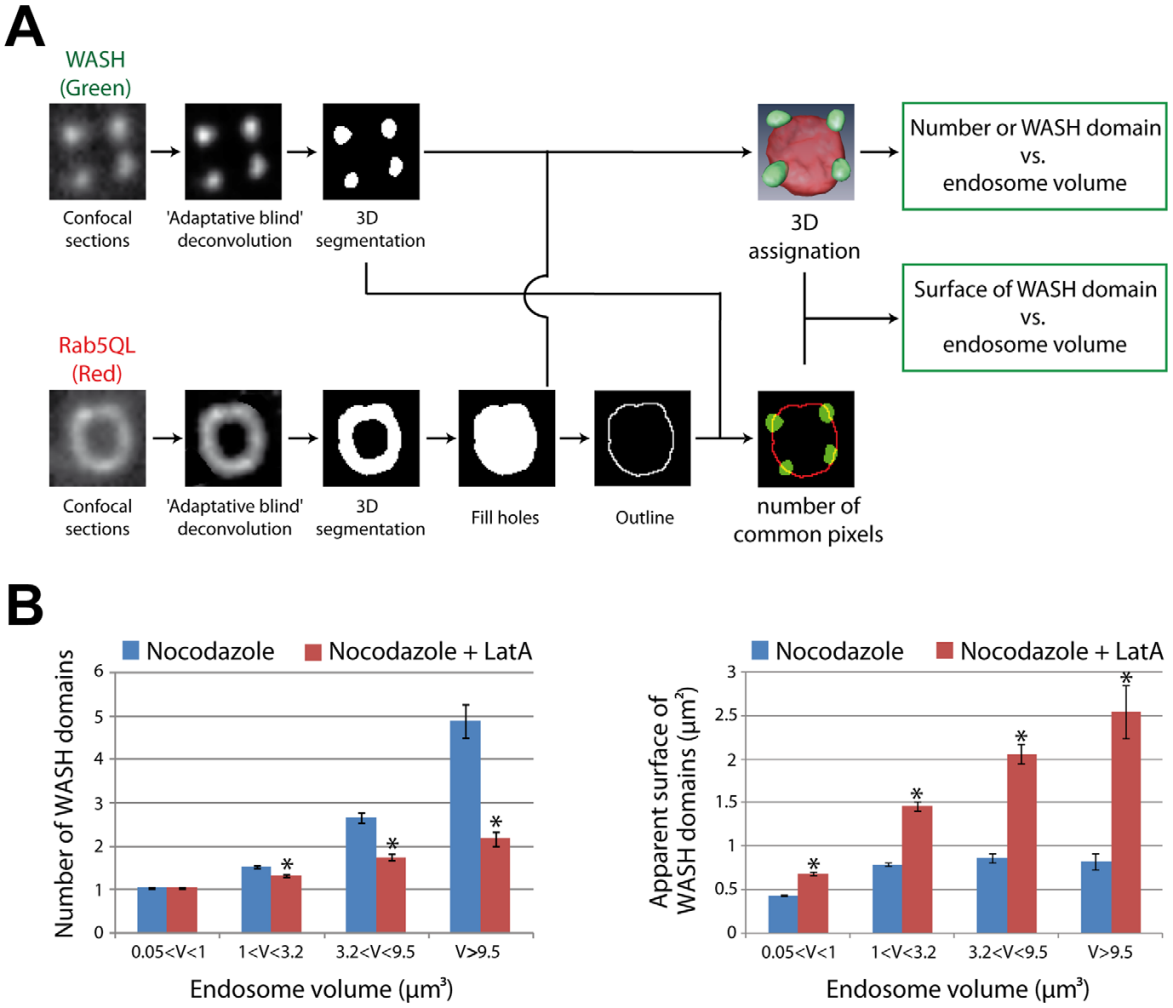
Quantification of the coalescence of WASH domains upon actin depolymerization. (A) Image processing workflow (see also Methods). Confocal slices were acquired for GFP-WASH and mCherry-Rab5Q79L channels and processed as in Fig. 2 B, except that the wavelet filter was substituted by an ‘Adaptative blind’ deconvolution step. (B) Image stacks were segmented in 3D for both channels (23 cells, 2179 endosomes for control; 22 cells, 1221 endosomes for LatA). The average number of WASH domains (± s.e.m.) and their average apparent surface (± s.e.m.) were plotted as a function of endosome volume. The average number of WASH domains increases with endosome volume (p<0.001 Kruskal-Wallis one way analysis of variance on ranks). Moreover, upon actin depolymerization, the number of WASH domains in large endosomes decreases (*: p<0.05 compared with control within the same volume bin, Kruskal-Wallis test followed by a Dunn pairwise comparison). Concomitantly, the surface occupied by WASH domains increases (* p<0.001 compared with control within the same volume bin, two way ANOVA followed by a Tukey pairewise comparison). Altogether, these data suggest that WASH domains coalesce upon actin depolymerization.

This analysis confirmed quantitatively that LatA treatment significantly shifts the distribution of endosome volumes, with a reduced number of normal sizes and the appearance of large endosomes above 1 µm^3^ (Fig. 2C, p<0.001, χ^2^ test). Moreover, this analysis showed that in the control situation, WASH domain intensity increases with the volume of the endosome (Fig. 2D). This is a ∼2-fold effect, when the smallest endosomes are compared to the largest ones. However, the LatA treatment induced a 3-fold increase of the WASH intensity, whenever endosomes of similar size are compared (Fig. 2D). As a proxy for WASH surface density, we calculated the apparent concentration of WASH by dividing the intensity of each WASH domain by the volume it occupies. This ‘apparent concentration’ of WASH is remarkably constant over the whole range of endosome sizes, but increases about 2-fold when actin is depolymerized by LatA (Fig. 2E). All the above observations were statistically significant (p<0.001, two way ANOVA followed by a Tukey Test for pairwise comparisons). Importantly, these quantitative results were also observed when CytoD was used instead of LatA to depolymerize actin (Fig. S2).

**Figure 8:**
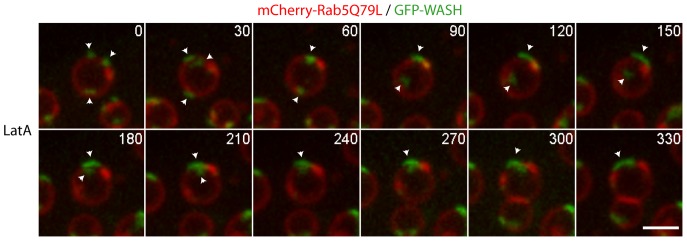
Direct observation of WASH domain coalescence in live cells upon actin depolymerization. Stable 3T3 cells expressing GFP-WASH and transiently expressing mCherry-Rab5Q79L were treated with 10 µM nocodazole for 1 h, then imaged by live spinning disk confocal microscopy. 12 planes separated by an increment of 0.4 µm were acquired at each time point, and z-projected. Elapsed time is in seconds. Scale bar: 2 µm. 0.2 µM LatA was added at the beginning of the movie. Arrowheads show individual WASH domains that fuse sequentially.

This increased WASH recruitment at the surface of endosomes was observed with endogenous WASH analyzed by immunofluorescence. One can argue that this effect of LatA is due to a better accessibility of WASH to antibodies, when actin networks are depolymerized. We therefore performed the same experiment in a stable 3T3 cell line that expresses GFP-WASH. This GFP-WASH construct rescues the defect associated with depletion of endogenous WASH [7]. The stable cell line expresses GFP-WASH at a level similar to that of endogenous WASH, because GFP-WASH replaces the endogenous WASH in its multiprotein complex (Fig. S3A). We observed similar behavior for GFP-WASH and its endogenous counterpart (Fig. S3B–D), ruling out the possible artifact of a better accessibility effect after LatA treatment.

**Figure 9:**
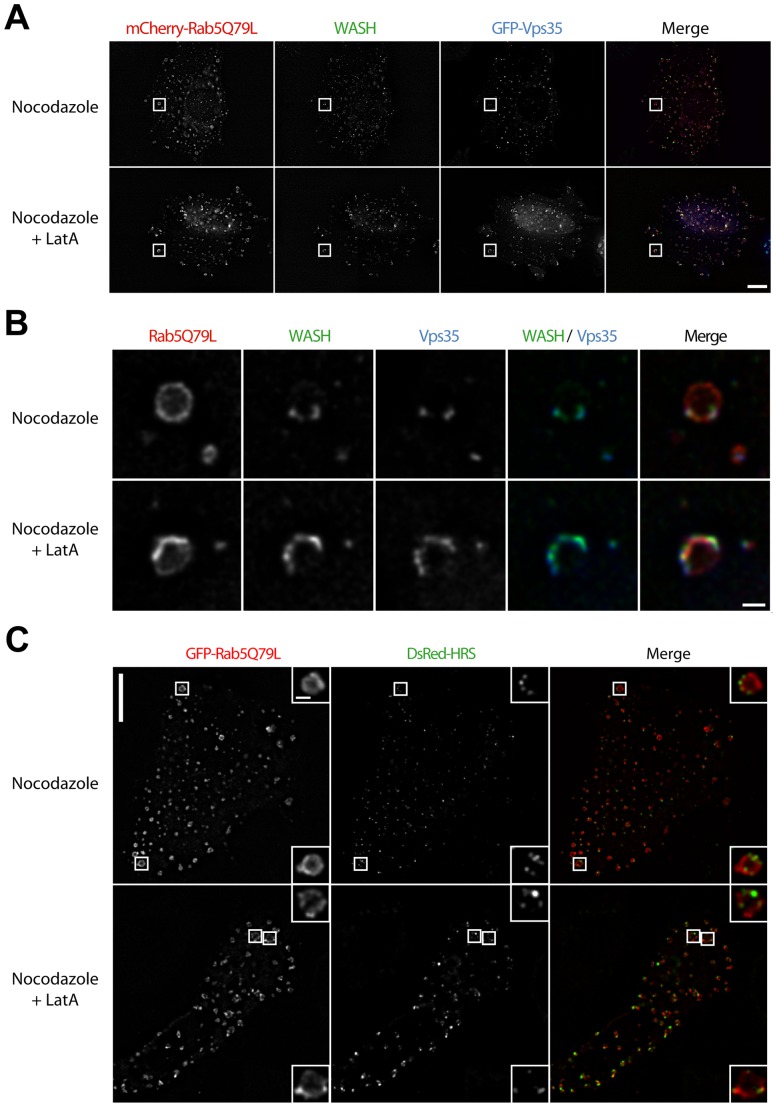
The domains formed by the Vps subcomplex of the retromer, but not HRS domains, coalesce like WASH domains. (A) Stable 3T3 cells expressing GFP-Vps35 were treated as in Fig. 6A, then processed for immunofluorescence using WASH antibody and observed by epifluorescence microscopy followed by deconvolution (single planes). Scale bar: 10 µm. (B) corresponds to the insets displayed in (A). Scale bar: 1 µm. Vps35 colocalizes with WASH and behaves similarly to WASH upon actin depolymerization. (C) 3T3 cells were transfected with GFP-Rab5Q79L and DsRed-HRS, and treated as above. Cells were fixed and observed by spinning disk confocal microscopy followed by deconvolution (single planes). Scale bar: 10 µm (1 µm in insets). HRS localizes to discrete domains at the surface of enlarged endosomes, whether or not actin polymerization is prevented.

**Figure 10:**
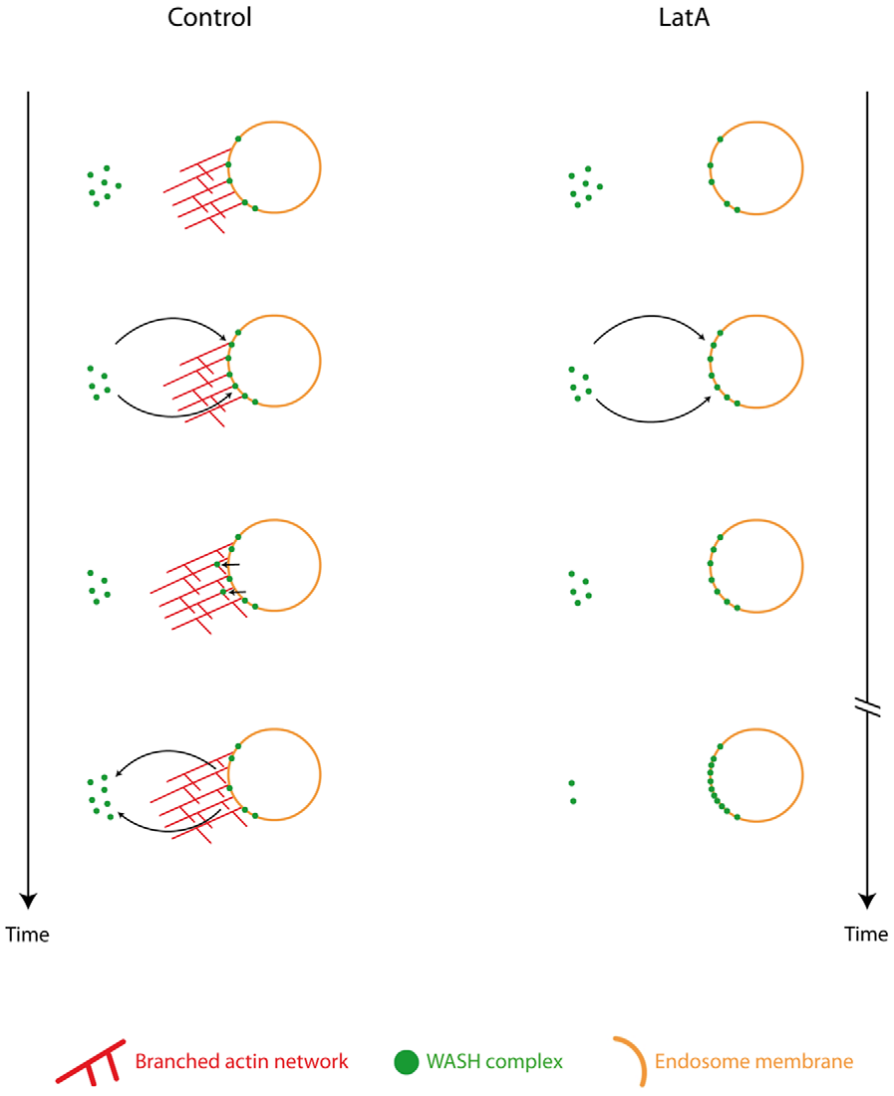
Interpretation for the role of actin on WASH dynamics on and off endosomes. To explain how actin depolymerization increases the steady state amount of WASH on endosomes and decreases the exchange of endosomal WASH for cytosolic WASH, we propose that actin branched networks promote the detachment of WASH from endosomes. For example, endosomal WASH may bind to the active Arp2/3 complex at branched junctions of the actin network, and detach from the endosome as the branched junction moves backward because of filament elongation.

Altogether, these image analyses suggested two conclusions: (i) large endosomes have larger WASH domains than smaller ones, but with a similar surface density of WASH, (ii) in contrast, actin polymerization negatively regulates the surface density of WASH in its domain.

To evaluate the dynamics of WASH when actin polymerization is impaired or not, we used Fluorescence Recovery After Photobleaching (FRAP) of GFP-WASH. WASH domains of similar size were bleached in control cells and in LatA treated cells. The bleached zone was large enough to encompass the whole endosome ensuring that fluorescence recovery was due to recruitment of cytosolic WASH and not to lateral diffusion (Movie S1). Recovery of fluorescence was monitored for 3 min (Movie S2 and Fig. 3A). To quantify the recovery, we tracked the bleached WASH domain, since its position fluctuates over time, and verified that the tracked centroid of WASH fluorescence remained over time onto the same endosome surface, which was visualized using the unbleached signal of Tf. More than 50 recovery curves were averaged in both cases (Fig. 3B). In control cells, WASH recovers to ∼60% of the initial fluorescence during the first minute. This plateau at 60% indicates that 40% of WASH molecules are immobile at this time scale. In sharp contrast, when actin is depolymerized, WASH recovers to only 20% after 3 minutes, corresponding to 80% immobile WASH. This result suggests that the exchange of WASH between cytosolic and endosome-bound pools strongly depends on the actin cytoskeleton.

However, LatA depolymerizes all actin networks, whether the Arp2/3 complex had nucleated them or not. We thus depleted the Arp2/3 complex to specifically impair the generation of branched actin networks. Transfection of 3T3 cells with siRNAs targeting p34Arc induced a strong decrease in the protein levels of p34Arc and p16Arc, suggesting depletion of the entire complex (Fig. 4A). Arp2/3 depletion reduced considerably Tf internalization, in line with the implication of the N-WASP-Arp2/3 pathway in clathrin-mediated endocytosis [26], [27]. We therefore used EEA1, which allowed us to detect early endosomes similarly in Arp2/3 depleted and non depleted cells. Arp2/3 depletion led to an increased intensity of GFP-WASH staining on EEA1 positive endosomes (Fig. 4B). We were able to quantify WASH on the whole population of early endosomes, upon microtubule depolymerization using the method described above. As for pharmalogical inhibition of actin polymerization, Arp2/3 depletion by two independent siRNAs led to a significant increase of GFP-WASH recruitment at the surface of endosomes, when endosomes of similar size were compared (Fig. 4C and D). Together, these experiments suggest that the dynamics of branched actin networks at the surface of endosomes increases the dynamics of WASH, the multiprotein complex that controls the formation of these same branched actin networks.

### Actin depolymerization promotes lateral coalescence of WASH domains at the surface of large endosomes

We often observed elongated GFP-WASH-positive structures on large endosomes upon Arp2/3 depletion (Fig. 5A). Such structures were also observed with endogenous WASH upon Arp2/3 depletion (Fig. 5B). The appearance of such elongated WASH structures upon actin depolymerization was previously noticed and attributed to endosome tubulation [9]. To verify whether these elongated structures were indeed membrane tubules, we imaged GFP-WASH in Arp2/3 depleted cells using time-lapse spinning disk confocal microscopy. In this experiment, we added fluorescent Tf in the medium, which provided us with a weak endosomal signal, because its internalization is impaired by Arp2/3 depletion. Strikingly, WASH positive structures on dimly Tf-stained endosomes did not exhibit the fluctuations expected for fine membrane tubules, but appeared rather as crescent-shaped domains at the surface of large endosomes (Fig. 5C, Movie S3). The same observation was made with a LatA treatment following Tf internalization (Movie S4, see also Fig. 1A). These WASH crescents on large endosomes contrast with the several discrete WASH domains detected when endosomes are enlarged by homotypic fusion through the expression of the active mutant of Rab5, Rab5Q79L [7], [40]. These observations suggested the idea that actin depolymerization induces coalescence of WASH domains.

To investigate this possibility, we examined the effect of LatA on endosomes, which were already enlarged by transient transfection with mCherry-Rab5Q79L. Confocal examination of LatA treated cells indeed revealed numerous examples of large endosomes, in which the number of WASH domains is reduced and their surface increased compared to control cells (Fig. 6A). 3D reconstructions confirmed this impression obtained from single confocal planes (Fig. 6B and Movie S5). We modified slightly our image analysis program to obtain the number of WASH domains per endosome and their apparent surface area (Fig. 7A). In the control situation, the number of WASH domains indeed increases with the endosome volume, from 1 for the smallest endosomes to about 5 for the largest ones (Fig. 7B). This result is in line with the observation that WASH localizes to one domain on small EEA1 endosomes, but to two domains on larger ones in HeLa cells [8]. The apparent surface that each WASH domain occupies saturates at about 0.8 µm^2^ for endosomes larger than 1 µm^3^. When LatA is applied, the increase in the number of WASH domains as a function of endosome volume is strongly diminished. For the largest endosomes, it culminates at 2 WASH domains per endosome, compared to 5 in the control situation. Concomitantly, the apparent surface that each WASH domain occupies increases steadily to 2.5 µm^2^ for the largest endosomes. All the above observations were statistically significant (see legend of figure 7).

Altogether, these image analyses suggested two conclusions: (i) large endosomes have more WASH domains than smaller ones; (ii) actin polymerization constrains the surface area of WASH domains below an upper limit.

To directly demonstrate that actin depolymerization induces coalescence of WASH domains, we sought to image the process after LatA addition. To alleviate the problem of out of plane WASH domains on enlarged endosomes, we acquired 12 z-planes by spinning disk confocal microscopy every 30 seconds, and performed a maximum intensity z-projection of the planes (Fig. 8 and Movie S6). This experiment showed that actin depolymerization induces WASH domain coalescence in a sequential manner whenever they meet.

We initially reported that WASH belongs to a stable multiprotein complex [7]. We thus investigated how another subunit of the WASH complex, namely FAM21, behaves upon LatA treatment. As expected, FAM21 colocalized with WASH both in control and LatA-treated cells (Fig. S4). This suggests that actin depolymerization does induce the coalescence of the whole WASH complex at the endosome surface and thus does not interfere with the stability of the complex.

We next examined whether coalescence upon actin depolymerization is a general behavior of endosomal domains or a specificity of WASH domains. The Vps35 subunit of the so-called ‘cargo selective subcomplex’ of the retromer was recently shown to bind directly to the FAM21 subunit of the WASH complex and to be required for the endosomal recruitment of the WASH complex [10], [41]. We thus thought to study whether retromer domains similarly coalesce upon actin depolymerization. We established a stable 3T3 cell line which expresses GFP-Vps35 and found that Vps35 and WASH colocalize in discrete domains at the surface of Rab5Q79L endosomes in control cells (Fig. 9A–B). Upon LatA treatment, Vps35 remained associated with the coalesced WASH domain. HRS, a component of the ESCRT-0 complex, also defined discrete domains on enlarged endosomes. However, these domains were distinct from the ones of WASH, and they did not coalesce upon actin depolymerization (Fig. 9C). These results suggest that domain coalescence upon actin depolymerization is a specific property of the domain containing both WASH and retromer complexes.

## Discussion

Using LatA mediated actin depolymerization, we have observed two effects on the Arp2/3 activator WASH at the surface of endosomes. The first one is an increased WASH staining on endosomes and the second one is the coalescence of WASH domains at the surface of large endosomes. We are confident that these two effects are not due to artifactual antibody staining, as they were similarly observed using a GFP-WASH fusion protein. These effects were best seen when endosomes were scattered using microtubule depolymerization. However, the increased WASH staining and the crescent shaped WASH domain were also observed without nocodazole, suggesting that these alterations of WASH distribution solely rely on the actin cytoskeleton. Indeed, Arp2/3 depletion produced the same phenotype, thus ruling out non specific effects of LatA treatment. We attempted to confirm the endosomal WASH enrichment that we have observed upon actin depolymerization using established procedures for membrane cytosol biochemical fractionation assays [42], [43]. However these attempts were not succesful. Several non-exclusive reasons might account for this discrepancy between microscopy and biochemical fractionations, such as the effect of dilution that displaces the equilibrium between these two pools or other exchanges between different membrane pools.

To accurately quantify these effects on WASH distribution upon LatA treatment, we had to develop automated image analysis techniques to assign each WASH domain to its correct endosome in 3D. Specifically, our method overcomes the acute problem of the heterogeneity of size and shapes of endosomes. This method helped us to accumulate sufficient data to prove that the two effects on WASH distribution we report here are indeed general effects independent of the variable sizes and shapes of endosomes. We anticipate that these programs, available on request, will be useful for future endosomal studies, for instance to quantify protein recruitment onto endosomes and endosomal protein contents in vivo.

Arp2/3 depletion phenocopies LatA treatment on WASH distribution, thus suggesting that WASH is controlled by the branched actin networks it generates through feedback mechanisms. Indeed, both treatments induce an accumulation of WASH on endosomes. Moreover, FRAP experiments on GFP-WASH have revealed that LatA treatment ‘freezes’ the dynamic exchange of WASH between cytosolic and endosomal pools. The lack of WASH recovery after photobleaching indicates that cytosolic WASH is less recruited to endosomes upon LatA treatment. There are two possible interpretations to explain this effect. Either the recruitment of WASH from the cytosol requires actin – however, it is difficult to envision in this case why the endosomal pool of WASH increases upon LatA treatment – or, it is the detachment of WASH from endosomes that requires actin. We favor this hypothesis, which is consistent with the increased endosomal pool of WASH upon actin depolymerization. This hypothesis is less intuitive as to why there is less recovery of WASH after photobleaching. It is likely that defective recovery of GFP-WASH in this hypothesis is due to the constant occupancy of endosomal binding sites by bleached WASH, which do not detach to return to the cytosol.

Such a feedback mechanism of actin networks on other Arp2/3 activators has been observed in different systems. Actin depolymerization increases the membrane pool of WAVE complex and freezes its dynamics in neutrophils [44]. Actin depolymerization also freezes the dynamics of N-WASP in the actin tails of Vaccinia virus [45]. For these reasons, we favor a model in which the Arp2/3 complex and its activator have intrinsic built-in dynamics rather than a specific biochemical pathway mediating this feedback regulation. One possible molecular scenario, explaining how the regulation of WASH dynamics may arise from its own activity, is depicted in Fig. 10. Active WASH molecules at the surface of endosomes activate Arp2/3 complexes, which generate branched actin networks. If the interaction between WASH and Arp2/3 is maintained long enough while the branched junction moves backwards, because of filament elongation, then one might understand how WASH, and by extension any Arp2/3 activator, can be actively detached from the membrane where it has been activated. Indeed, in vitro evidence exists for a transient tripartite interaction implicating the actin network, the Arp2/3 complex and its activator N-WASP [46]. In the cell, WASH molecules from the lamellipodium were also found by speckle microscopy to undergo a retrograde movement like the Arp2/3 complex at the branched junction [47].

We have also observed that branched actin networks control the lateral distribution of WASH domains at the surface of endosomes, since actin depolymerization induces coalescence of the WASH domains. This spontaneous coalescence of WASH domains is reminiscent of the behavior of lipidic domains at the surface of artificial vesicles [48], [49]. The WASH complex directly binds to lipids through its FAM21 subunit, which has a broad specificity for negatively charged lipids [7], [24]. This interaction however seems insufficient to account for membrane recruitment of the WASH complex, since recent experiments showed the cargo selective complex of the retromer was required to recruit the WASH complex to the endosomal surface through an interaction between FAM21 and Vps35 [10], [41]. The ability of the WASH complex to interact with lipids might promote lipid reorganization after it has been recruited to the endosomal surface through the retromer. Once WASH is at the surface of the endosome, it is possible that it recognizes specific lipids and hence that WASH behavior reflects the behavior of the underlying lipids. These ideas need to be confirmed by the identification of the precise lipids that the WASH complex recognizes at the surface of endosomes and the development of specific probes thereof. A crucial step in the dissection of these mechanisms will be the ability to reconstitute these phenomena in vitro with purified machineries at the surface of model membranes.

Our hypothesis to explain WASH coalescence is centered on a platform containing specific lipids clustered by the WASH complex. The WASH platform would have an intrinsic coherence that is independent of actin, and a long-range organization that depends on the branched actin network it is associated with. The short-range coherence accounts for the coalescence of two platforms when they meet at the surface of endosomes and for the fact that WASH molecules remain in clusters without actin. Without such a short-range coherence, we would expect WASH molecules diffusing all over the surface of endosomes, which we did not observe with or without actin. This short-range coherence might arise from WASH induced lipid repartitioning and domain formation, as previously described for some viral or bacterial lipid binding proteins [50], [51]. The long-range organization provided by the branched actin network is required to understand why the platform cannot grow above an upper limit and prevent coalescence of WASH platforms when they encounter at the surface of endosomes. One can imagine that the WASH mediated branched actin network can grow only until a definite shell-like architecture that prevents further addition of WASH-lipids units is reached. This long-range organization argues for an active role of the actin cytoskeleton in lateral compartmentalization of endosomal membranes, which is crucial for sorting processes.

The WASH complex might promote the scission of transport intermediates by several mechanisms, which collaborate. First, it recruits dynamin, directly or indirectly through the cortactin-positive branched actin network it generates [7]. Second, it probably favors the so-called line tension driven mechanism of membrane scission through an ability to organize a proteo-lipidic platform. Lipid repartitioning and domain formation is not only favored by protein binding to lipids, but also by membrane curvature, which is high in the tubule concentrating cargoes [51], [52], [53]. Line tension, a force that develops at the interface between lipid domains, favors constriction and ultimately scission of the membrane tubule [48], [49], [52], [54]. The actin cytoskeleton was recently demonstrated to perform lipid reorganization and dynamin-independent scission of membrane tubules induced by Shiga toxin in HeLa cells [55]. The WASH complex thus has the required properties to coordinate these two mechanisms for the scission of endosomal transport intermediates.

## Materials and Methods

### Cell treatments

Flip-In 3T3 cells (Invitrogen) and 3T3 GFP-WASH stable cells were handled and transfected as previously described [7] with the following modifications for Arp2/3 depletion: 40 nM of On-Target Plus siRNA (p34 duplex #1: sense AGGAAGCGCUGUCGACCGA; p34 duplex #2: sense GGUAAUGAGUUGCAGGUAA Dharmacon, Lafayette, Colorado) were transfected once, retransfected 2 days later and analyzed after 2 more days. Antibodies and constructs were previously described in [7], except pAb targeting EEA1 (Cell Signaling Technology, Beverly, MA), Ds-Red-HRS plasmid (gift of Dominique Lallemand, Institut Curie, Paris) and GFP-Rab5Q79L plasmid (gift of Philippe Chavrier, Institut Curie, Paris). Transferrin (Tf) uptake was performed as described [7]. For drug treatment, nocodazole (Sigma, St. Gallen, Switzerland), Latrunculin A (Cayman Chemicals, Ann Harbor, MI) and Cytochalasin D (Merck, Darmstadt, Germany) were used in the internalization medium in the presence of Tf.

Full length human Vps35 was amplified by PCR using a template provided by L. Johannes (Institut Curie, Paris) and cloned into a modified pCDNA5/FRT/V5-His (Invitrogen) vector, in which the CMV promoter was replaced by a EF1α/HTLV chimera promoter and which tags the ORF by a N-terminal eGFP [7]. Stable 3T3 transfectants obtained by homologous recombination at the FRT site were obtained as previously described [7].

### SDS-PAGE and Western Blot

Whole cell lysates were prepared by resuspending cells directly in SDS-PAGE loading buffer. Viscosity of samples after boiling was reduced using Benzonase (Sigma). SDS-PAGE was performed using NuPAGE 4–12% Bis-Tris gels (Invitrogen) transferred onto nitrocellulose membranes according to the manufacturer's instructions. Western blots were revealed using HRP coupled antibodies, Supersignal kit (Pierce) and a Fuji LAS-3000 (Fujifilm).

### Immunocytochemistry and image acquisition

For standard imaging of fixed samples, 3T3 cells were plated for 2 h onto glass coverslips coated with 50 µg/ml fibronectin (Sigma) before each experiment, fixed in PBS-3% paraformaldehyde, permeabilized in PBS containing 0.05% saponin, then processed for indirect immunofluorescence using standard techniques. Samples were then mounted in ProLong® Gold antifade reagent (Invitrogen).

To preserve the structure of enlarged endosomes, cells co-expressing mCherry-Rab5Q79L and GFP-WASH or GFP-Vps35 were plated for 2 h onto fibronectin-coated surfaces (glass plates, from Iwaki, or μ-slides, from IBIDI, Martinsried, Germany), fixed in PBS-3% paraformaldehyde for 10 min at 37°C, processed for immunofluorescence as described above, then washed and imaged in PBS. For time-lapse microscopy, cells were plated for 2 h onto fibronectin-coated glass (Iwaki) or plastic (μ-Dish, IBIDI) plates and imaging was performed in DMEM medium supplemented with 10 mM Hepes (Invitrogen).

Epifluorescence microscopy was performed as previously described [7] using an AxioObserver Z1 microscope (Carl Zeiss, Jena, Germany) equipped with a 63× NA 1.4 oil immersion objective, an additional 1.6× lens and an Orca-R^2^ camera (Hamamatsu, Hamamatsu City, Japan).

Confocal sections were acquired using two custom spinning disk setups based on TE2000-U or TI-Eclipse inverted microscopes (Nikon, Tokyo, Japan), equipped with temperature control chambers (The Cube, Life Imaging Services, Basel, Switzerland or Okolab, Quarto, Italy), 100× oil immersion objectives (NA 1.45 or 1.49), CSU22 or CSU-X1 spinning disk heads (Yokogawa Electric Corporation, Tokyo, Japan), CoolSnap HQ2 or Evolve EM-CCD cameras (Photometrics, Tucson, AZ), and operated by Metamorph 7.1.4 or 7.6 (Molecular Devices, Sunnyvale, CA), respectively. The TE2000-U was equipped with a piezoelectric translator mounted on the objective to control the position of the confocal plane (Physik Instruments, Karlsruhe, Germany). For dual-color live imaging, channels were acquired sequentially and, for each time point, several planes were acquired and projected to compensate the exit of endosomes from the confocal plane. Analyses of images and movies were performed using Metamorph and ImageJ softwares (http://rsb.ingo.nih.gov/ij/).

When indicated, 3D deconvolution was performed using the ‘Adaptative Blind’ algorithm of Autoquant ×2 software (MediaCybernetics, Bethesda, MD). 3D reconstructions were performed using Amira software (Visage Imaging Inc., San Diego, CA) on deconvolved images. Movies were edited with Adobe Premiere software.

### 3D analysis of endosome volume and WASH domains

For preliminary quantification (Fig. 1), confocal slices (0.2 µm increment) were acquired for WASH and Transferrin (Tf) channels. Background of images was homogenously subtracted in ImageJ. In order to enhance the detection of small and dim objects, stacks were then processed using a wavelet ‘à trous’ filter (‘ImproveKymo’ ImageJ plugin, developed by Fabrice Cordelière, Institut Curie, Orsay). 3D segmentation was then performed using ‘3D object counter’ ImageJ plugin (Bolte and Cordelières, 2006), keeping the intensity threshold constant for the WASH channel. Measurement of WASH fluorescence intensity over the segmented regions was redirected to background-subtracted images. Single peripheral Tf-positive endosomes were manually selected and their corresponding WASH integrated intensity averaged.

For automated analysis on scattered endosomes (Figs. 2 and 4, S2 and S3), the image processing workflow is presented in Fig. 2B. The first processing steps were identical to those of the preliminary analysis. Since Tf is a membrane marker, large endosomes were detected as hollow structures that were subsequently filled for accurate volume measurement (‘Fill Holes’ ImageJ function). Each WASH domain was then assigned, in 3D, to its proper endosome using a custom MATLAB code (see paragraph below), and the total WASH fluorescence intensity of domains associated to the endosome was measured. Due to unavailability of one microscopy setup, we had to use a different one to acquire data quantified in Figs. 2, S3 and Figs. 4, S2. We thus normalized the volumes of both datasets to obtain the same endosome volume bins between experiments. The normalization factor was calculated based on the average endosome size in control cases conditions, using the LatA dataset (Fig. 2) as a reference.

To assign each WASH domain to its endosome, we developed a custom-made code in MATLAB (Mathworks, Natick, MA). First, a common pixel yellow stack is generated from the segmented images of both green (WASH) and red (Tf) channels. This stack is then segmented and each yellow region is assigned to its red and green object, from which the association between the red and the green regions is deduced. Two geometrical methods were used to assign each Yellow object to its Green or Red object. First, a ‘Minimum Distance’ method finds the closest Green and Red objects for each Yellow object, based on the 3D-distance between geometrical centers. Second, a ‘Bounding Box’ method assigns a Yellow object to a Green (or Red) object if the fluorescence centroid of the Yellow object is contained within the minimum bounding box containing the Green (or Red) object. We excluded all the objects for which both methods gave different results (less than 2% of total WASH domains). This MATLAB code is available on request.

For cells expressing mCherry-Rab5Q79L (Fig. 7), image analysis was conducted essentially as above, except that the wavelet ‘à trous’ filter was replaced by a deconvolution step (‘Adaptative Blind’ Autoquant algorithm). Apparent surface of each subdomain was measured by counting the number of common pixels between the outline of the Rab5Q79L endosome, obtained using the ‘outline’ ImageJ function, and the solid WASH domain. Since WASH domains are usually detected on the side and not on the top or on the bottom of endosomes, we converted pixel number in a surface using a pixel size of 126×200 nm^2^ (XY-spacing×Z-spacing).

### Histograms and statistics

Data were plotted using Matlab and statistical tests were performed using Matlab or Sigmastat (Systat software). Endosome volume distributions were compared with the Chi-2 test while pooling volume bins superior to 1.4 to have more than 5 endosomes per bin. The preliminary analysis of the influence of actin depolymerization on the average WASH domain intensity (Fig. 1B) was analyzed using a one way ANOVA after a Log10 transformation. The influence of both actin depolymerizating treatments and endosome volume on the average intensity of WASH domains, their ‘apparent concentration’ or their ‘apparent surface’, was analyzed after a Log10 transformation using a two-way ANOVA with treatments and volumes as parameters. In all cases, pairwise comparisons were performed with the Tukey Test using an α factor of 0.05. Since the number of WASH domains per endosome is a discrete value, the influence of actin depolymerization on domain number (Fig. 7B) was analyzed using a non parametric Kruskal–Wallis one-way analysis of variance on ranks and pairwise comparisons were performed with the Dunn Test using an α factor of 0.05.

### FRAP

FRAP experiments were performed on the second spinning disk confocal microscope setup, using a 100× NA 1.40 PlanAPO objective and a FRAP module equipped with 491 and 642 nm lasers (Roper Scientific SARL, Evry, France). Circular regions centered on WASH domains (10 px in diameter) were selected (∼5–10 regions/cell), and bleached for 50 ms using the 491 nm laser. Recovery was monitored on a single plane for 3 min (1 min at 1 frame/s, then 2 min at 0.5 frame/s), acquiring GFP-WASH and Alexa647-Tf channels sequentially using a µs-AOTF switch. FRAP analysis was performed with ImageJ using custom-made macros unless stated otherwise. First, the background was homogenously subtracted and the global photobleaching was corrected over the entire image. Then, WASH domains were manually tracked using the ‘MTrackJ’ ImageJ plugin developed by Eric Meijering. Finally, the fluorescence of WASH domains was integrated at each time point in a circular region (6 px in diameter) centered on the fluorescence centroid. Each WASH domain trajectory was examined and compared to the trajectory of its counterpart in the Tf channel. Aberrant trajectories and recoveries were discarded.

## Supporting Information

Figure S1 LatA treatment induces close to complete depolymerization of branched actin networks associated with WASH-positive endosomes. (A) 3T3 cells were treated as in Fig. 2A, and pre-extracted before fixation, as described in Derivery et al (2009). Cells were then processed for immunofluorescence using WASH and cortactin antibodies, and observed by epifluorescence microscopy (single planes). Scale bar: 10 µm. (B) corresponds to the insets shown in (A). The fluorescence intensities of WASH and cortactin were measured along linescans drawn on the endosomes (white lines). Under LatA treatment, WASH signal increases, whereas cortactin signal decreases, indicating the disappearance of the endosomal branched actin network.
(TIF)

Figure S2 CytoD treatment increases endosomal WASH similarly to LatA. (A) 3T3 cells were loaded with fluorescent Tf until equilibrium, then treated with 10 µM nocodazole in the continuous presence of Tf for 1 h, then treated with 1 µM CytoD or carrier in the presence of nocodazole and Tf for 30 min. Cells were then processed for immunofluorescence and observed as in Fig. 2. Scale bar: 10 µm. (B–C) Image stacks (25 cells, 3324 endosomes for control; 28 cells, 5203 endosomes for Cyto D) were processed and presented as in Fig. 2D–E, after normalization of endosomes volumes (*: p<0.001 compared with control within the same volume bin, ANOVA2 followed by a Tukey pairwise comparisons test). (B) The intensity of GFP-WASH domains increases when actin is depolymerized. (C) The ‘apparent concentration’ of GFP-WASH in domains increases upon actin depolymerization and does not depend on endosome volume.
(TIF)

Figure S3 Actin depolymerization increases the amount of GFP-WASH associated with endosomes. (A) Stable 3T3 cells expressing GFP or GFP-WASH were analyzed by Western Blot using indicated antibodies. GFP-WASH overexpression is limited. GFP-WASH replaces endogenous WASH in the stable cell line. (B) Cells expressing GFP-WASH were treated as in Fig. 2A, then fixed and imaged by spinning disk confocal microscopy (single planes). Scale bar: 10 µm. (C–D) Image stacks (14 cells, 1639 endosomes for control; 21 cells, 3056 endosomes for LatA) were processed and presented as in Fig. 2D–E (*: p<0.001 compared with control within the same volume bin, ANOVA2 followed by a Tukey pairwise comparisons test). (C) The intensity of GFP-WASH domains increases when actin is depolymerized. (D) The ‘apparent concentration’ of GFP-WASH in domains increases upon actin depolymerization and does not depend on endosome volume.
(TIF)

Figure S4 FAM21 colocalizes with WASH, whether or not actin is depolymerized. (A) Stable 3T3 cells expressing GFP-WASH were treated as in Fig. 6A, then processed for immunofluorescence using FAM21 antibodies and observed by epifluorescence microscopy followed by deconvolution (single planes). Scale bar: 10 µm. (B) corresponds to the insets displayed in (A). Scale bar: 1 µm. FAM21, another subunit of the WASH complex, colocalizes with WASH and forms crescents with WASH upon actin depolymerization.
(TIF)

Movie S1 FRAP analysis of GFP-WASH dynamics. Several WASH domains were bleached in a nocodazole-treated cell. The bleached regions are indicated by circles in the 6 frames before bleaching. The insets show WASH domains (green) and the Tf-positive endosomes (red). Frame rate: 20 images per second. Scale bar: 10 µm.
(MOV)

Movie S2 Actin depolymerization inhibits WASH recovery on endosomes after photobleaching. This movie corresponds to Fig. 3. Frame rate: 20 images per second.
(MOV)

Movie S3 Depletion of the Arp2/3 complex induces the formation of enlarged endosomes harboring a crescent-shaped WASH domain. Figure 5C is extracted from this movie. Frame rate: 1 image per second, 2 planes with an increment of 0.5 µm z-projected per time point. Scale bar: 10 µm.
(MOV)

Movie S4 Actin depolymerization by LatrunculinA induces the formation of enlarged endosomes harboring a crescent-shaped WASH domain. 3T3 cells stably expressing GFP-WASH were treated as in Fig. 2A and imaged by live spinning disk confocal microscopy. During the 1-min course of this movie, large endosomes displaying large and static crescent-shaped WASH domain at their surface are observed. Frame rate: 1 image per second, 2 planes with an increment of 0.3 µm z-projected per time point. Scale bar: 10 µm (1 µm in insert).
(MOV)

Movie S5 3D reconstructions of WASH domains on Rab5Q79L-enlarged endosomes in the presence or not of LatrunculinA. Figure 6B is extracted from this movie.
(MOV)

Movie S6 Direct observation of WASH domain coalescence upon actin depolymerization. Two examples are shown. Figure 8 is extracted from the left movie. Scale bar: 2 µm.
(MOV)
