# Supplementary figures and images for: Actin Polymerization Controls the Organization of WASH Domains at the Surface of Endosomes
Source: PLoS One. 2012 Jun 21;7(6):e39774. doi: 10.1371/journal.pone.0039774 (PMC3380866; doi:10.1371/journal.pone.0039774)

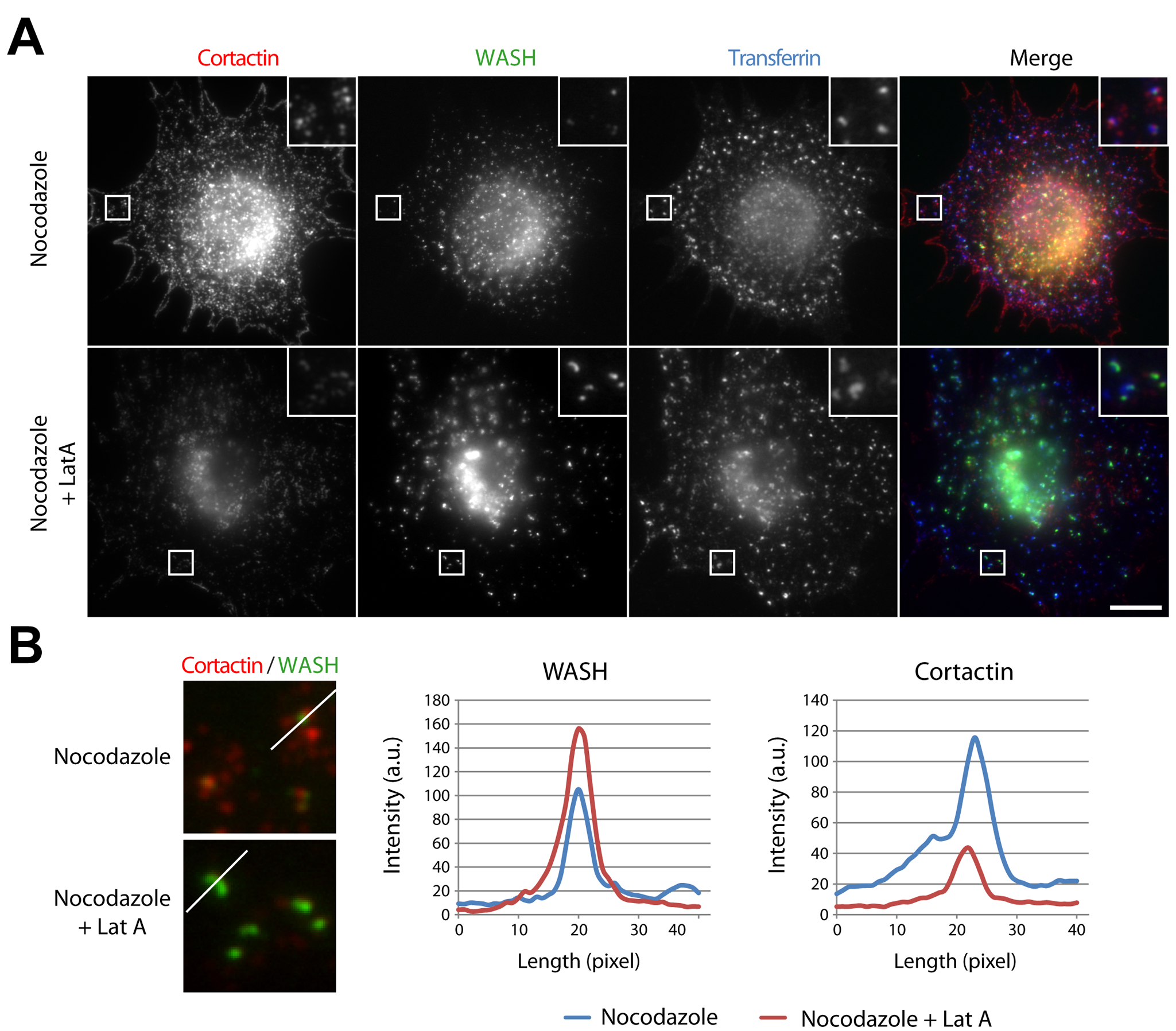

Supplement: Figure S1 — LatA treatment induces close to complete depolymerization of branched actin networks associated with WASH-positive endosomes. (A) 3T3 cells were treated as in Fig. 2A, and pre-extracted before fixation, as described in Derivery et al (2009). Cells were then processed for immunofluorescence using WASH and cortactin antibodies, and observed by epifluorescence microscopy (single planes). Scale bar: 10 µm. (B) corresponds to the insets shown in (A). The fluorescence intensities of WASH and cortactin were measured along linescans drawn on the endosomes (white lines). Under LatA treatment, WASH signal increases, whereas cortactin signal decreases, indicating the disappearance of the endosomal branched actin network. (TIF) [file pone.0039774.s001.tif]

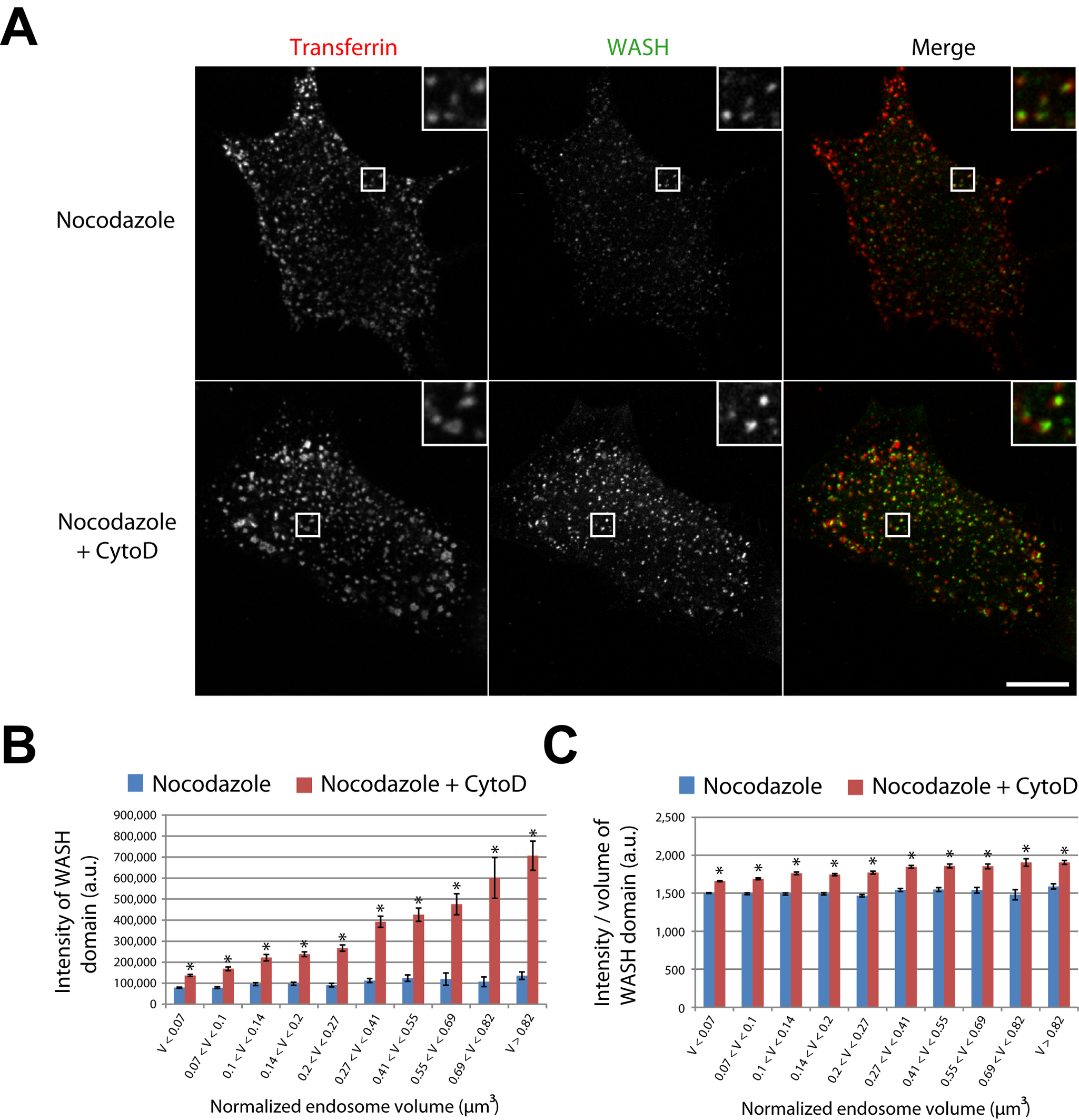

Supplement: Figure S2 — CytoD treatment increases endosomal WASH similarly to LatA. (A) 3T3 cells were loaded with fluorescent Tf until equilibrium, then treated with 10 µM nocodazole in the continuous presence of Tf for 1 h, then treated with 1 µM CytoD or carrier in the presence of nocodazole and Tf for 30 min. Cells were then processed for immunofluorescence and observed as in Fig. 2. Scale bar: 10 µm. (B–C) Image stacks (25 cells, 3324 endosomes for control; 28 cells, 5203 endosomes for Cyto D) were processed and presented as in Fig. 2D–E, after normalization of endosomes volumes (*: p<0.001 compared with control within the same volume bin, ANOVA2 followed by a Tukey pairwise comparisons test). (B) The intensity of GFP-WASH domains increases when actin is depolymerized. (C) The ‘apparent concentration’ of GFP-WASH in domains increases upon actin depolymerization and does not depend on endosome volume. (TIF) [file pone.0039774.s002.tif]

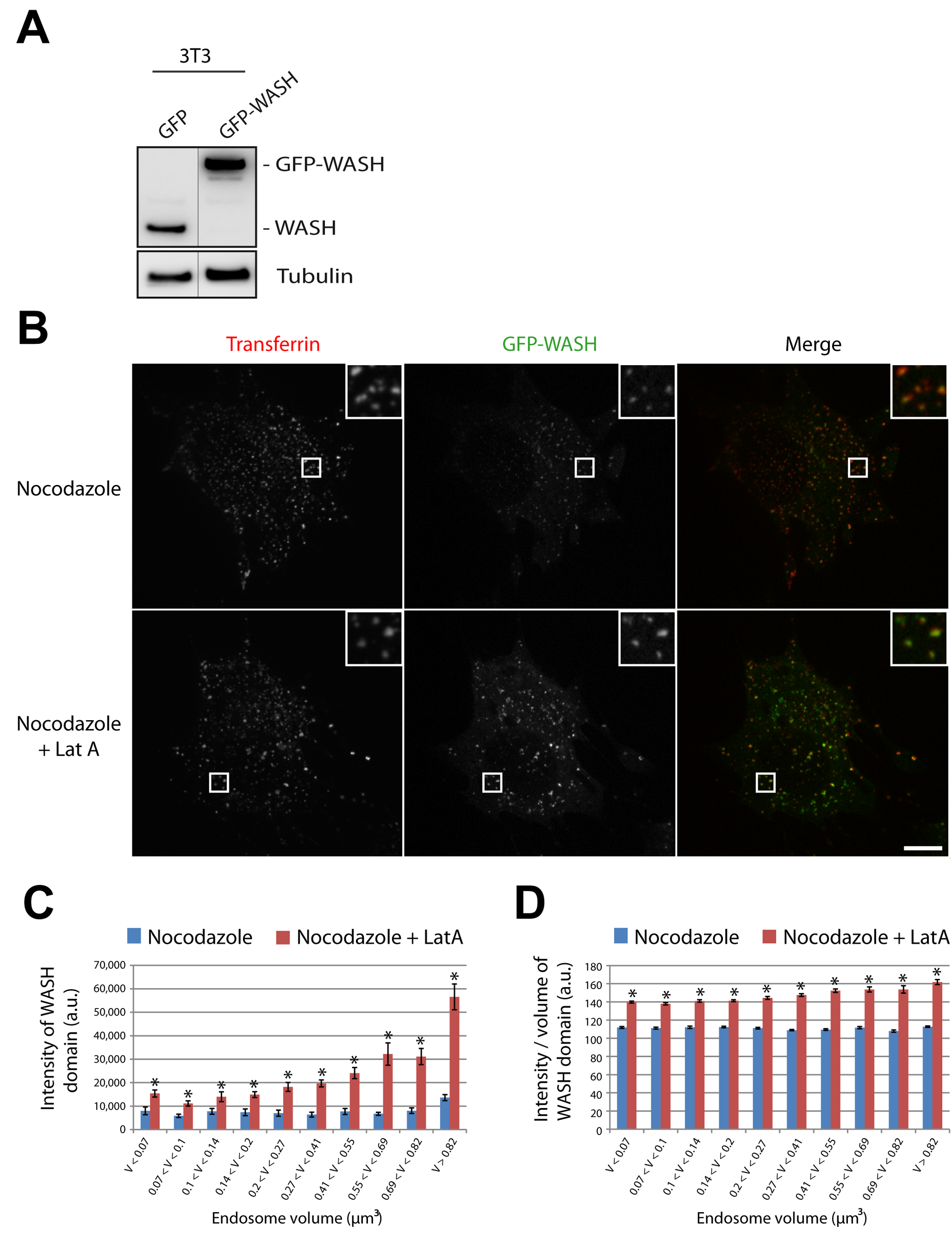

Supplement: Figure S3 — Actin depolymerization increases the amount of GFP-WASH associated with endosomes. (A) Stable 3T3 cells expressing GFP or GFP-WASH were analyzed by Western Blot using indicated antibodies. GFP-WASH overexpression is limited. GFP-WASH replaces endogenous WASH in the stable cell line. (B) Cells expressing GFP-WASH were treated as in Fig. 2A, then fixed and imaged by spinning disk confocal microscopy (single planes). Scale bar: 10 µm. (C–D) Image stacks (14 cells, 1639 endosomes for control; 21 cells, 3056 endosomes for LatA) were processed and presented as in Fig. 2D–E (*: p<0.001 compared with control within the same volume bin, ANOVA2 followed by a Tukey pairwise comparisons test). (C) The intensity of GFP-WASH domains increases when actin is depolymerized. (D) The ‘apparent concentration’ of GFP-WASH in domains increases upon actin depolymerization and does not depend on endosome volume. (TIF) [file pone.0039774.s003.tif]

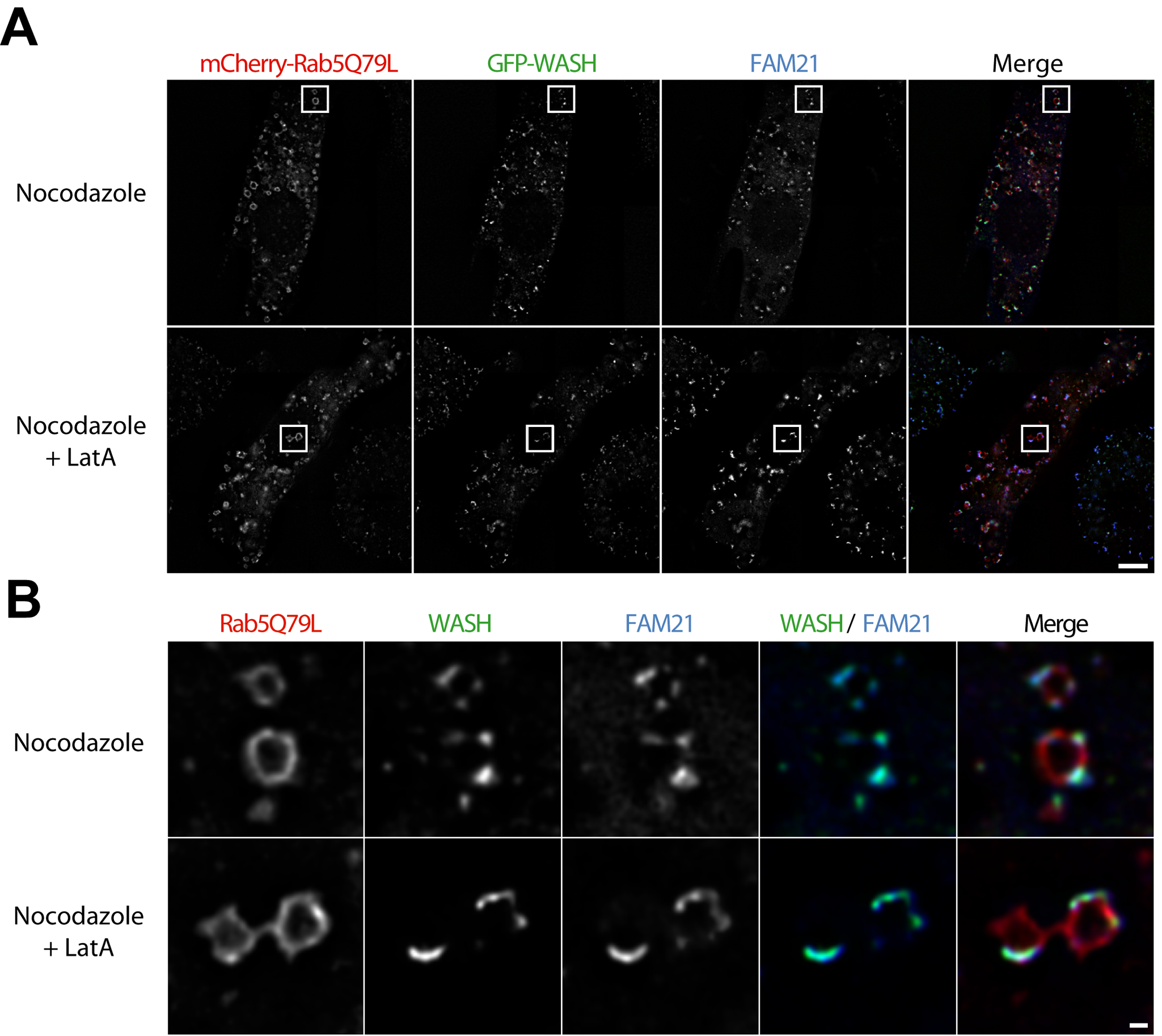

Supplement: Figure S4 — FAM21 colocalizes with WASH, whether or not actin is depolymerized. (A) Stable 3T3 cells expressing GFP-WASH were treated as in Fig. 6A, then processed for immunofluorescence using FAM21 antibodies and observed by epifluorescence microscopy followed by deconvolution (single planes). Scale bar: 10 µm. (B) corresponds to the insets displayed in (A). Scale bar: 1 µm. FAM21, another subunit of the WASH complex, colocalizes with WASH and forms crescents with WASH upon actin depolymerization. (TIF) [file pone.0039774.s004.tif]
